# Morphological Features of the Ovaries During Oogenesis of the Oriental Fruit Fly, *Bactrocera dorsalis*, in Relation to the Physiological State

**DOI:** 10.1673/031.012.14401

**Published:** 2012-12-09

**Authors:** Ming-Yi Chou, Ronald F. L. Mau, Eric B. Jang, Roger I. Vargas, Jaime C. Piñero

**Affiliations:** ^1^ University of Hawaii at Manoa, College of Trop. Agric. & Human Resources. 3050 Maile Way, Honolulu, HI 96822; ^2^ U.S. Pacific Basin Agricultural Research Center, USDA-ARS, P.O. Box 4459, Hilo, HI 96720; ^3^ Lincoln University of Missouri, Cooperative Research and Extension, Allen Hall 212, 900 Chestnut St., Jefferson City, MO 65102

**Keywords:** age, egg load, follicular relics, parity, physiological status

## Abstract

Determination of physiological state in insects is useful in furthering the understanding of how insect behavior changes with age. Central to this determination is the identification of characters that allow assessment of physiological age. While non-destructive measures produce the most desired outcomes, internal markers may be more diagnostic and reliable. In this study, key morphological characters during previtellogenesis through vitellogenesis and ovulation were assessed as markers to determine physiological states of the oriental fruit fly, *Bactrocera dorsalis* (Hendel) (Diptera: Tephritidae). Ovary length and width, ovarian index (length × width), and egg load of laboratory-reared *B. dorsalis* females recorded daily from eclosion up to 80 days old suggested significant differences in the ovarian index and egg load between females from each oogenesis stage. Parity status determined by the presence of follicular relics was found to provide high-accuracy classifications for *B. dorsalis* females. The presence of follicular relics with distinct morphological features provides a reliable identification tool to determine the physiological state of wild female oriental fruit fly. The potential applications of this technique to identify the physiological age of female fruit flies to study behavioral attributes in their natural habitat, and also the potential applications in relation to field control, are discussed.

## Introduction

In female oriental fruit flies, *Bactrocera dorsalis* (Hendel) (Diptera: Tephritidae), ovarian development represents a time line from previtellogenesis to complete egg development for the purpose of male fertilization. Changes of the ovary's morphological characters from oogenesis and ovulation are key signifiers of physiological age for long-lived female tephritids (Carey 2001; Kendra et al. 2006). Changes in the length and width of the ovaries as a result of yolk protein accumulation in the follicles are followed by a concomitant expansion of egg chambers during vitellogenesis. The end of the egg production process is marked by the formation of follicular relics (corpora lutea) at the basal part of the ovarioles after ovulation (Fletcher et al. 1978). Nonrecurring changes of ovary morphology from continuous egg production in conjunction with accumulations of follicular relics are two main indicators of physiological age for dipteran pest species of medical and economic importance (Nation 1972; Kapatos and Fletcher 1984; Magnarelli et al. 1984; Gryaznov 1995).

The association between the age of females and their physiological status is the foundation of many control strategies developed for tephritid fruit fly pest management (Aluja 1994; Prokopy 2003; Vargas et al. 2010). Age-related demographic parameters have been used for predicting field establishment of pest populations and for timing augmentative biological control programs (Vargas and Ramadan 2000). The physiological age of an individual modulates the behavioral ontogeny of a female tephritid in response to inherited genetic traits, food quality and quantity, temperature, and other environmental factors (Prokopy et al. 1994; Vargas et al. 1997; Wang et al. 2009). In this context, physiological age involves behavioral modification of response threshold to stimuli with time. Females switch from food-foraging to host-searching oriented behaviors as they approach sexual maturity; this switch is a result of modifying threshold to odors in the environment (Siderhurst and Jang 2006; Siderhurst and Jang 2010). Monitoring programs with food or host attractants may thus provide quantitative information relevant to the age structure of a population.

Various methods have been evaluated for age determination in insects, including approaches based on adult morphology, physiology, and biochemistry (Hayes and Wall 1999; Hugo et al. 2008). Accumulation of pterin fluorescent pigment compounds over time in the head capsule of *Anastrepha ludens* (Loew), *Bactrocera Cucurbitae* (Coquillett), and *Ceratitis capitata* (Wiedemann) was measured to determine age dependent linear regressions (Hayes and Wall 1999). This method needs to factor in increasing pterin level due to ambient temperature, light level, and protein feeding (Hugo et al. 2008). For sterile insects released into the environment, the degree of wing abrasion gives a useful estimate of survival age (Dyck et al. 2005). Cuticle deterioration in age determination has the advantage of being independent of factors affecting the reproductive system, such as protein availability, although other factors, for example temperature or habitat, may influence fly activity and hence the rate of degradation. Physiological age-grading systems are useful, particularly for female insects, where physiological (rather than chronological) age determines key life history traits such as lifespan, fecundity, and foraging behaviors (Tyndale-Biscoe 1984). The study of physiological age distribution gives insights for understanding population biology and describing behaviors of tephritid populations. Knowledge of the physiological age distribution of a particular tephritid population holds a crucial key to successful pest management. Tephritid pests, such as olive fly (*Bactrocera oleae* Gmelin), walnut fly (*Rhagoletis juglandis* Cresson), cherry fruit fly (*R. indifferens* Curran), and Chinese citrus fruit fly (*B. minax* Enderlein), have been reported to synchronize their ovarian development to coincide with host-fruit ripening (Delrio and Cavalloro 1977; Kapatos and Fletcher 1983; Messina et al. 1991; Carsten and Papaj 2005; Dorji et al. 2006). In orchards, these monophagous tephritids were effectively controlled when management efforts began after the capture of the first egg bearing female.

Many tropical tephritid pests are polyphagous fruit flies, which seek oviposition sites according to available host fruits in the environment. Effective pest management depends on determining the movement of mature females. Studies have found that the response of *Anastrepha suspensa* (Loew) to proteinaceous odors and distance of attraction varied with sexual maturity of the females (Kendra 2009; Kendra et al. 2010). Physiological age and parity have not yet been investigated in the oriental fruit fly, a major economic pest in tropical and subtropical fruit-producing areas in Asia and Hawaii (Seo et al. 1982; Vargas et al. 1983; Mau et al. 2007). Suitable tools are needed in order to assess the physiological age of a targeted population for optimal timing of application of food-based attractants. The objectives of this study were (1) to describe in detail the basic reproductive morphology as well as the overall development of the reproductive system of female *B. dorsalis* through time, (2) to describe the morphological features of oogenesis stages based on ovary length and width, and (3) to assess the characters of the parous females by examining the presence of retained mature eggs, corpora lutea, and other morphometric features of the ovaries

## Materials and Methods

### Flies

*B. dorsalis* were obtained from cultures maintained under laboratory conditions for > 300 generations at the Pacific Basin Agricultural Research Center, Honolulu, Hawaii, and maintained in a room at 24 ± 2° C and 50 ± 5% RH, with a photoperiod of about 16:8 L:D (Vargas et al 1984). Newly emerged adults were supplied with sugar and enzymatic yeast hydrolysate protein (United States Biochemical Corp., http://www.usbweb.com/) mixed in water in a 3:1 ratio *ad libitum*. Four cages with 100 pairs of females and males each were sample cohorts. Ten female individuals were dissected daily from emergence to day seven to record the morphometric changes during the first gonotrophic cycle, and then every 10 days for 80 days after the sampled cohort grew fully developed ovaries, which occurred at day seven. Fresh papaya was provided in the cage for oviposition from day seven onwards, when all sampled flies were bearing fully developed eggs.

As oogenesis is asynchronous in dacine fruit flies, the classification of ovarian development was based on the condition of the most advanced follicles when assigning individuals to a particular oogenesis stage (Fletcher et al. 1978; Klowden 2007). Stage 1 and 2 were part of the previtellogenic phase. Ovaries with no visible follicle cells presented were categorized as Stage 1. Stage 2 began when the developing oocytes entered the vitellarium region once it had been completely surrounded by the follicular epithelium. The nurse cells were located at the anterior end of the follicle, and the oocyte at its posterior end (Stage 3). The size of the terminal follicle increased rapidly during the vitellogenesis phase (Stage 4) as yolk protein was being transported into the developing oocyte. The follicular epithelium and nurse cells degenerated at the end of vitellogenesis after the secretion of chorion was completed (Stage 5). As ovulation proceeded, the egg was ejected from the ovariole while the empty follicle shrunk up and collapsed, forming a spherical body known as corpus luteum. Stretched, empty ovarioles and the presence of follicle relics at the calyx and lateral oviduct areas marked the parous phase (Stage 6).

Flies were placed in a 0° C freezer for 30 minutes prior to examination. The reproductive system was extracted from the abdomen under a stereomicroscope equipped with an ocular micrometer at 10-20x magnification. Dissection was conducted by grasping the abdominal cuticle along the mid-dorsal line with a fine forceps and tearing open the cuticle, exposing the ovaries in the abdominal cavity. Ovaries were removed and excised from the ovipositor and adhering tissues and rinsed with phosphate buffered saline (pH = 7 PBS). Ovary samples were stained with aqueous neutral red (dimethyl diaminophenazine chloride; toluylene red) saline solution (0.001%) for 30–40 seconds (Gryaznov 1995). The biometric parameters recorded included the length of the ovaries from the anterior end of the germarium to the calyx area and the width of the ovaries taken from the anterior end of the vitellarium. The Ovarian index, one of the parameters used to determine the stage of oogenesis, was obtained by multiplying ovary length by ovary width. Parous females were identified by the presence of follicular relics, which often were of light yellow color after the neutral red stain. The egg load was determined by counting the number of chorionated fully-developed oocytes in the egg chambers.

### Data analysis

One-way analysis of variance (ANOVA) was performed to compare changes in key ovarian characters and egg loads (SAS Institute 1998). The morphometric data of ovary length and width were square-root (x + 0.5) transformed prior to analysis. Data of ovarian index were log (x + 1) transformed prior to analysis. Tukey's HSD was performed to determine the difference of morphometric data between each oogenesis stage. Student's *t*-test was performed for two samples to determine the differences between the two measurements. The correlation between the morphometric parameters, ovary length, ovary width, and egg load were tested with Pearson correlation at *p* = 0.05.

## Results

**Figure 1.**
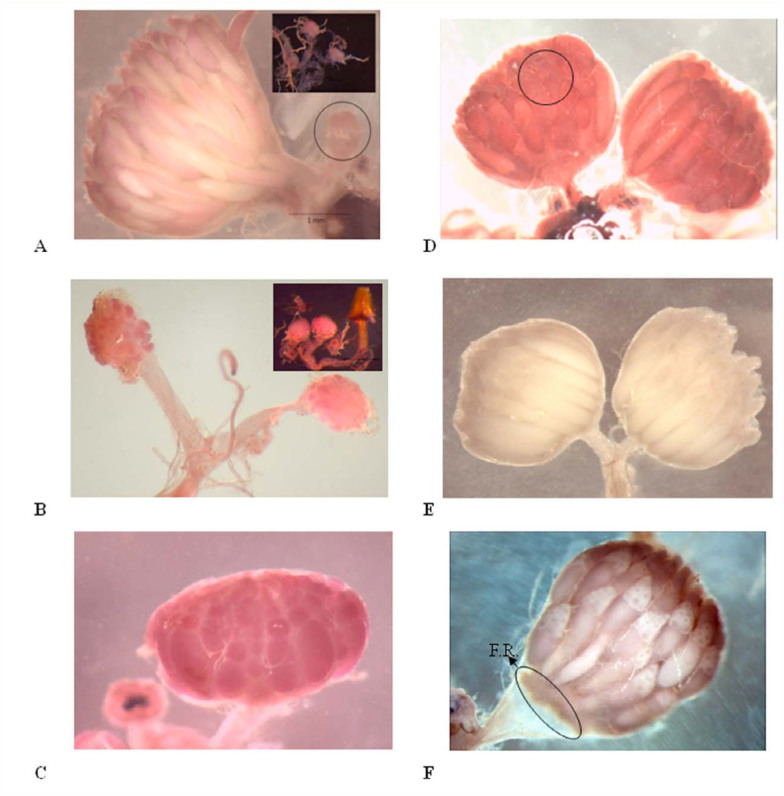
Stages of ovarian development in female *Bactrocera dorsalis*. Stage 1 (A, black circle) and 2 (B) represent previtellogenesis development. Stage 3 (C) marks the initiation of vitellogenesis, and 4 (D) indicates late vitellogenesis, at which point the yolk occupies more than half the follicle and nurse cells (black circle) occupy the anterior end of the oocyte. The presence of the first mature oocyte, characterized by an intact chorion with a reflective surface, indicates the beginning of stage 5 (E). Stage 6 (F) denotes parous females at the onset of oviposition, with the presence of follicular relics (F.R.) at base of the ovary (circle). High quality figures are available online.

Table 1 presents the morphometries of ovarian development in female *B. dorsalis*. The first oogenesis process was completed by seven days after adult eclosion. Stages 1 and 2 were the pre-vitellogenesis phase of the ovarian development. Each oocyte at the pre-vitellogenesis phase lacked a visible egg chamber. Stage 1 lasted for the first three days after emergence, with the ovaries approximately equal in length and width (Figure 1A). The vitellarium was not distinguishable from the lateral oviduct in Stage 1. As the developing oocytes moved down the ovariole and entered the vitellarium region between day three and four, the vitellarium area gradually became visible (Figure 1B). The length of the ovarioles averaged 0.4 ± 0.01 mm for Stage 1 and 0.6 ± 0.03 mm for Stage 2. Developing oocytes increased the ovary length significantly from Stage 1 to Stage 2 with no significant change in the width.

**Table 1.**
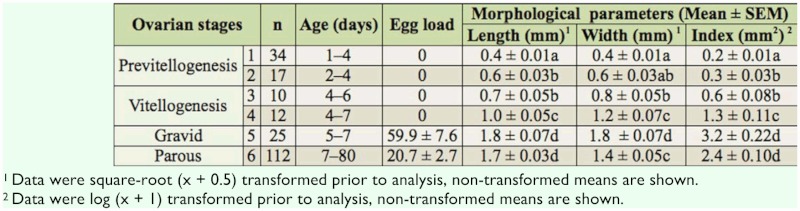
Ovarian characters, chronological age range and egg load at each developmental stage of *Bactrocera dorsalis*. Means within a column followed by the same letter are not significantly different (Tukey's HSD test (*p* = 0.05)).

Stage 3 (Figure 1C) was characterized by the onset of vitellogenesis, with nurse cells occupying the anterior end of the terminal follicle. There was a rapid transition between Stage 3 and 4 in four to six day old females once yolk began accumulating. The length and width of Stage 4 ovaries were significantly greater than both the pre-vitellogenesis phase and Stage 3. Stages 1–4 comprised the classes of nulliparous females (Table 1). The size of ovaries increased significantly from the growth of egg chambers during vitellogenesis. The duration of the vitellogenesis phase was on average 3 days from Stage 3 to the first batch of fully developed eggs. Nurse cells degenerated once the vitellogenesis was completed, and the process of forming chorion eggshells began.

**Figure 2.**
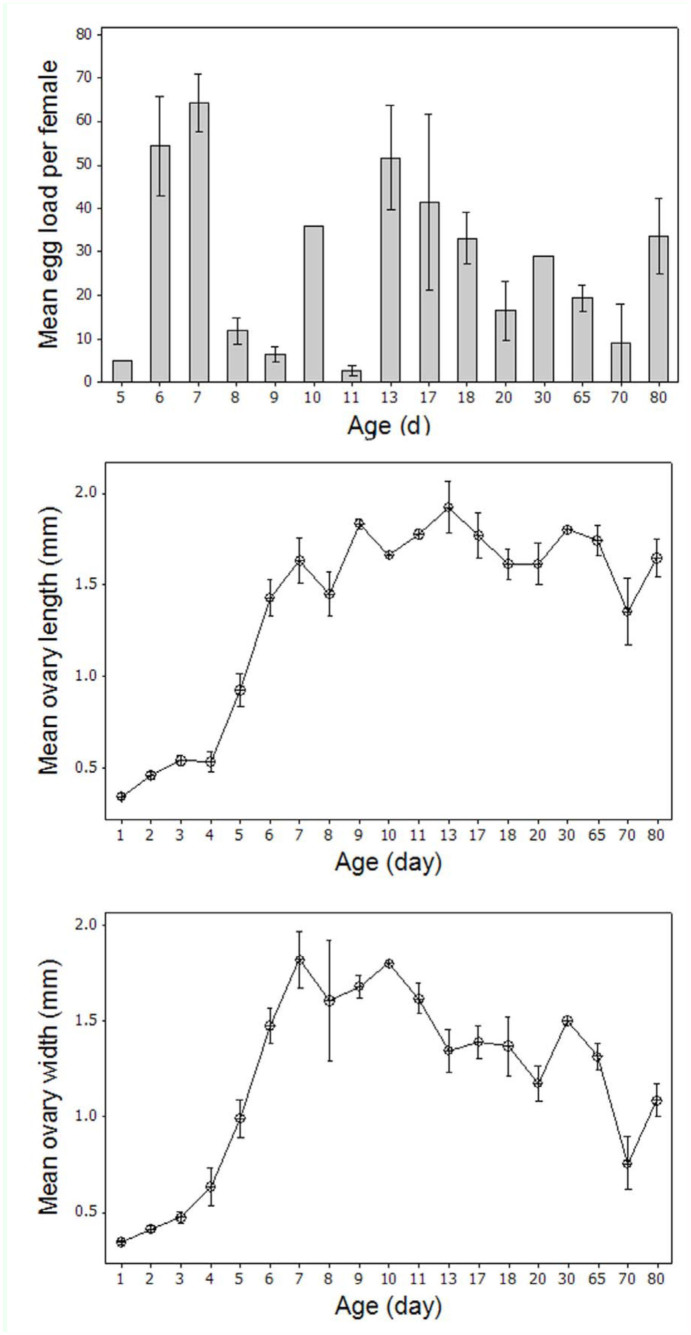
Egg load and ovary measurements (means and standard errors) of *Bactrocera dorsalis* five to 80 days old. High quality figures are available online.

The mature oocytes with chorion shells first appeared in the sampled females (Stage 5) as early as day five (Figure 1E). The egg load increased significantly between day five and day seven during the development of the first egg batch (Figure 2A), and fully developed ovaries were recorded in all samples by day seven. Egg load was correlated to ovary width (Pearson correlation = 0.40; *p* < 0.001) and length (Pearson correlation = 0.61; *p* < 0.001). The first oviposition marked the transition to Stage 6 (Figure 1F), as determined by the presence of follicular relics (corpora lutea) formed after the release of the terminal follicles. The follicular relics accumulated and the calyx became swollen with increasing chronological age. One possible source of error in the determining the stage of an ovary was that a mature female might be recorded as a Stage 3 if she happened to be sampled in the interval between the laying of a complete batch of eggs and the maturation of the next. Empty ovaries with stretched and straightened vitellarium section were found in 20% (n = 22) of the dissected parous females aged between eight and 80 days old. The index, length, and width of empty ovaries from parous females were significantly different compared to females at Stage 3. Once ovulation was initiated, the ovary width varied according to the egg load, but the length did not change significantly. Stage 6 was characterized by an overall decline in egg load (Figure 2A) and ovary width (Figure 2B) with increasing age. The average egg load in females older than eight days (Stage 6) was significantly lower (*t* = 4.14, *p* < 0.0001, df =32) than Stage 5 females (Figure 2A). In Stage 6, the egg load of parous females was an average of 20 (± 5.6 S.E.) eggs per female throughout the observation, regardless of age. Developmental asynchrony increased in secondary oogenesis with aging. Despite this increase, ovary width and ovarian index (Figure 2C) were significantly greater in Stage 6 than in Stage 4. Follicular relics were found in 98% of the ovipositing females and were absent only in some females that were at the beginning of deploying their first egg batch.

## Discussion

The overall objectives of this study were to identify the morphometric parameters for each oogenesis stage and to identify characters to identify parous female *B. dorsalis*. The six stages of ovarian development proposed for *B. cacuminata* (Raghu et al. 2003) and *B. oleae* (Fletcher et al. 1978) fit the stages reported here for *B. dorsalis*. The morphometry of the ovary increased significantly between each oogenesis stage during the first gonotrophic cycle whereas few changes were found in the parous females. The presence of corpora lutea and the ovary length were two key characters to identify parous females. The morphometric index was significantly different between parous and nulliparous females.

The ovaries of the first oogenesis phase in *B. dorsalis* initially increased in length and subsequently in width during the first seven days after eclosion. Our results support the seven to ten day (at 24 ^°^C) preoviposition period previously recorded for the laboratory strain of *B. dorsalis* (Foote and Carey 1987; Vargas et al. 1997). Of the four characters examined, ovary length was the most reliable indicator for pre-vitellogenesis and vitellogenesis during the developmental stage. The vitellogenesis phase was marked by the appearance of nurse cells at the anterior end of the developing oocyte, and lasted until the complete development of chorion eggshell. A large amount of yolk protein was deposited into the developing oocyte during this stage, which caused a rapid change in the ovarian structure. Characters of the terminal follicles, in combination with the ovarian index, provided accurate identification for the stages of oogenesis and were consistent with the findings of Kendra et al. (2006).

Once a mature egg is produced, the next distal follicle begins to mature, regardless of the stage of the proximate follicles in the other ovarioles. Laboratory and wild *B. dorsalis* have similar reproductive cycles once the female reaches maturity (Vargas et al. 1984; Vargas and Carey 1990). The rapid development in the following oocytes therefore resulted in an increasingly asynchronous egg development in each ovariole. The follicular relics of *B. dorsalis* observed in this study appeared as clumps of cells in the calyx (Figure 6F). Individual follicular relics were visible by stretching the tissue with a fine needle. However, the heterogenic number of follicular relics between the ovarioles, due to developmental asynchrony, resulted in the increased difficulty to determine the exact number of gonadotrophic cycles. The two-class age-grading system widely used in field studies to determine the age structure of hematophagous dipterans, such as *Anopheles* and *Culex* mosquitoes, and the screw worm (*Cochliomyia hominivorax* (Coquerel)), are also applicable to *B. dorsalis* (Hayes and Wall 1999). The pre-oviposition development time in *B. dorsalis* colonies derived from wild flies was longer, with a larger range in variation, than the laboratory strain (Arakaki et al. 1984; Foote and Carey 1987), suggesting that morphological characters are suitable candidates in determining the physiological age of feral populations. This age-grading technique was applied to determine the parity status of field captured *B. oleae*, *B. cacuminata*, and *B. dorsalis* (Fletcher et al. 1978; Raghu et al. 2003). Based on comparisons of the morphological characters examined in this study, follicular relics and ovary length are reliable indicators to determine the age structure of *B. dorsalis*.

Physiological states (i.e., nutritional state, mating status, etc.) coordinated with oogenesis influence females’ foraging behaviors (Jang and Light 1991; Prokopy et al. 1991; Prokopy et al. 1995; Jang et al. 1998). One of the main tasks for a newly eclosed female is to forage for the nutrients required for egg production. A surge in protein feeding was recorded in *B. tryoni* at this stage (Meats and Leighton 2004). Behavioral studies have confirmed that female tephritids with developing ovaries have a stronger response to proteinaceous odors compared to mature females, which respond more strongly to host-fruit odors (Prokopy et al. 1991; Nigg et al. 1995; Cornelius et al. 2000; Rull and Prokopy 2000). Physiological changes triggered by mating consequentially alter female behaviors from food- and mate-oriented olfactory behaviors to a strong preference for host-fruit stimuli. Fluctuations of the egg load with age suggest alternating between food seeking and oviposition-site seeking behaviors in order to obtain the protein needed for egg development (Kendra et al. 2006). The physiological age of flies collected from food-based traps may provide more complete information for monitoring purposes. From an applied perspective, the classification of ovarian developmental stages in conjunction with assessment of egg load and parity status will facilitate the evaluation of the age structure of a fly population responding to specific lures in field trapping studies. Detailed behavioral studies are required to determine the effects of egg load on food-foraging behavior of female *B. dorsalis*, including its relationships with host-seeking and proteinaceous food-seeking decisions. The proposed classification system based on *B. dorsalis* in this study has applications for both laboratory and field research based on the observed characters of ovarian development as indicators for the female physiological age. The method described to characterize the parity status of *B. dorsalis* is the main key to determine the target physiological age group for control using food-based attractants in natural environments.
